# Adjuvants for cancer mRNA vaccines in the era of nanotechnology: strategies, applications, and future directions

**DOI:** 10.1186/s12951-024-02590-6

**Published:** 2024-06-02

**Authors:** Lei-Ming Cao, Yi-Fu Yu, Zi-Zhan Li, Nian-Nian Zhong, Guang-Rui Wang, Yao Xiao, Bing Liu, Qiu-Ji Wu, Chun Feng, Lin-Lin Bu

**Affiliations:** 1 State Key Laboratory of Oral & Maxillofacial Reconstruction and Regeneration, Key Laboratory of Oral Biomedicine Ministry of Education, Hubei Key Laboratory of Stomatology, School & Hospital of Stomatology, Wuhan University, Wuhan, 430079 China; 2 Department of Oral & Maxillofacial - Head Neck Oncology, School & Hospital of Stomatology, Wuhan University, Wuhan, 430079 China; 3 Department of Radiation and Medical Oncology, Hubei Key Laboratory of Tumor Biological Behavior, Hubei Provincial Clinical Research Center for Cancer, Zhongnan Hospital of Wuhan University, 169 Donghu Road, Wuhan, 430071 China; 4 Department of Gynecology, Maternal and Child Health Hospital of Hubei Province, Tongii Medical College, Huazhong University of Science and Technology, Wuhan, China

**Keywords:** mRNA cancer vaccine, Adjuvant, Drug delivery system, Nanotechnology

## Abstract

Research into mRNA vaccines is advancing rapidly, with proven efficacy against coronavirus disease 2019 and promising therapeutic potential against a variety of solid tumors. Adjuvants, critical components of mRNA vaccines, significantly enhance vaccine effectiveness and are integral to numerous mRNA vaccine formulations. However, the development and selection of adjuvant platforms are still in their nascent stages, and the mechanisms of many adjuvants remain poorly understood. Additionally, the immunostimulatory capabilities of certain novel drug delivery systems (DDS) challenge the traditional definition of adjuvants, suggesting that a revision of this concept is necessary. This review offers a comprehensive exploration of the mechanisms and applications of adjuvants and self-adjuvant DDS. It thoroughly addresses existing issues mentioned above and details three main challenges of immune-related adverse event, unclear mechanisms, and unsatisfactory outcomes in old age group in the design and practical application of cancer mRNA vaccine adjuvants. Ultimately, this review proposes three optimization strategies which consists of exploring the mechanisms of adjuvant, optimizing DDS, and improving route of administration to improve effectiveness and application of adjuvants and self-adjuvant DDS.

## Introduction

Since the outbreak of coronavirus disease 2019 (COVID-19) in early 2020, severe acute respiratory syndrome coronavirus 2 has spread globally, resulting in over 250 million confirmed cases [1]. In the fight against COVID-19, mRNA vaccines have emerged as a prominent solution. These vaccines offer significant advantages over traditional vaccine technologies, including higher production efficiency and enhanced safety. Moderna, a leading entity among mRNA vaccine developers, rapidly identified the antigenic sequence of the virus and produced the first mRNA-1273 vaccine within just 45 days. This vaccine later demonstrated a 94.1% efficacy rate in a phase III clinical trial, underscoring the promising potential of mRNA vaccine technology for future infectious disease responses [2]. The mRNA vaccine has emerged as a vital tool in humanity’s arsenal against the novel coronavirus. Beyond their application in viral infections, mRNA vaccines are also being explored for their potential in cancer treatment. Several clinical trials involving mRNA-based cancer vaccines have yielded promising outcomes, highlighting the potential of mRNA vaccines in oncology. This development points to a broader scope of application for mRNA technology, potentially revolutionizing the approach to cancer treatment [3, 4].

**Fig. 1:**
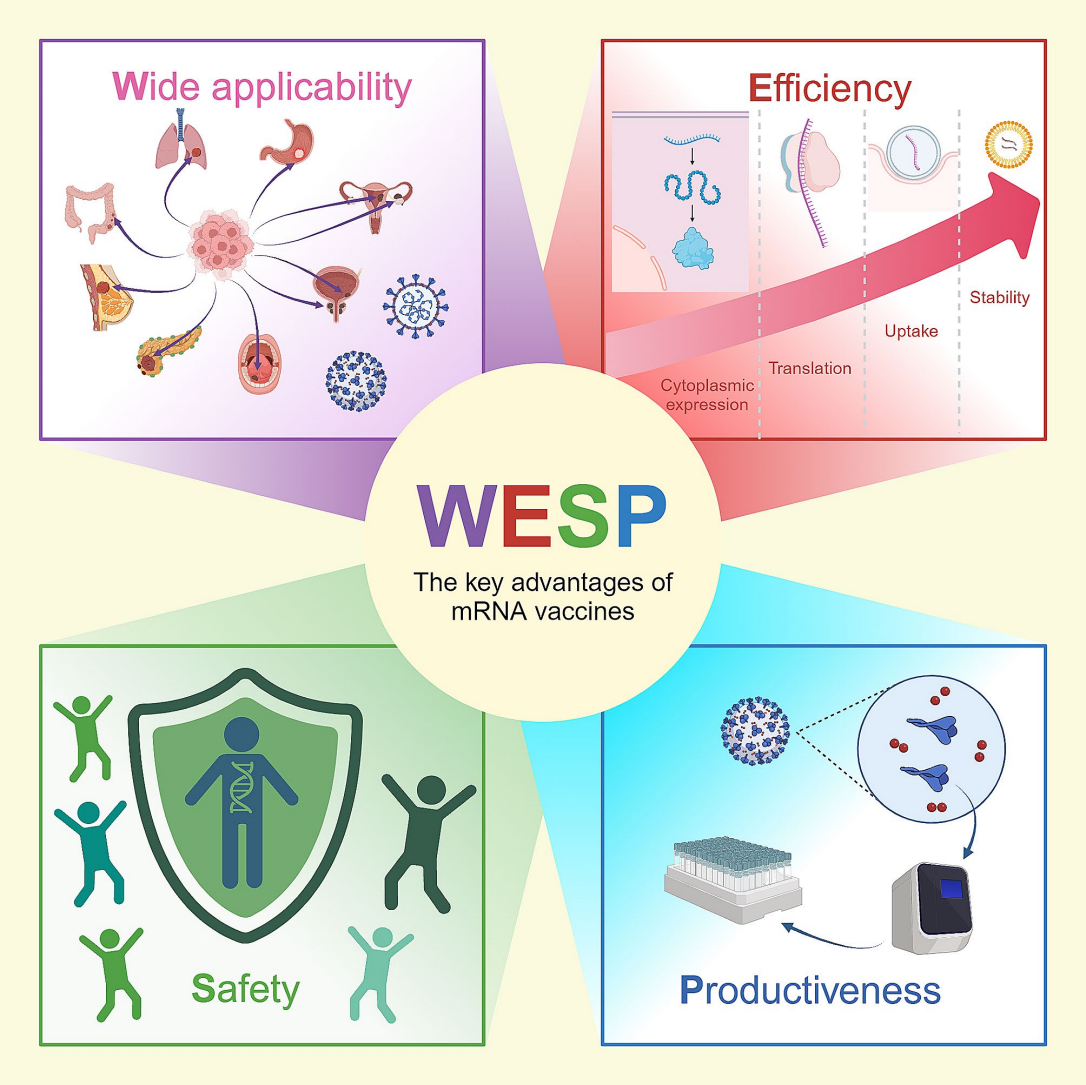
“WESP”-the key advantages of mRNA vaccines. The remarkable success of mRNA vaccines can be attributed to several key advantages which can be summarized by the acronym “WESP”: “W”: Wide applicability, “E”: Efficiency, “S”: Safety, “P”: Productiveness

The remarkable success of mRNA vaccines can be attributed to several key advantages, we summarized them by the acronym “**WESP**” (Fig. 1): (1) **Wide** applicability. mRNA vaccines can encode almost any protein and facilitate post-translational modifications within cells. This capability reduces immunogenicity while ensuring the functionality of protein products, leading to significant breakthroughs in treating various diseases [5–7]. (2) **Efficiency**. Appropriate modification and optimization of sequence can significantly improve mRNA stability and translation efficiency. Currently, there is already an efficient drug delivery systems (DDS) that can achieve rapid uptake and cytoplasmic expression of mRNAs [8–10]. (3) **Safety**. Unlike DNA vaccines, the mRNA platform is non-infectious and non-integrating, eliminating the risk of infection or gene insertion [11]. (4) **Productiveness**. Once the genome sequence of a pathogen is known, mRNA encoding the antigenic protein can be swiftly designed and produced. This was exemplified by the rapid development of mRNA vaccines for COVID-19. Furthermore, the high yield from in vitro transcription not only ensures rapid production but also makes the process cost-effective and scalable [2].

Aforementioned attributes collectively underscore the effectiveness and potential of mRNA vaccines as a pivotal tool in modern medicine, capable of addressing both infectious diseases and complex conditions like cancer. The mRNA vaccines offer a promising approach to cancer treatment by their ability to encode tumor-related antigens and elicit an immune response. The core principle of mRNA cancer vaccines involves transporting transcripts that encode for tumor-associated antigens or tumor-specific antigens into the cytoplasm of host cells, particularly antigen-presenting cells (APCs). This capability allows the immune system to recognize and target cancer cells effectively, potentially transforming cancer therapy by providing a highly specific and adaptive treatment option [12–14]. Currently, mRNA cancer vaccines made significant achievements in the treatment of prostate cancer. The prostate cancer vaccines CV9103, developed by Curevacs (Germany), has already undergone phase I/II clinical trials, during which it was demonstrated to be well tolerated and to elicit a favorable immune-activation [15]. In addition, in the phase I trial of a novel personalized mRNA neoantigen vaccine, it stimulated high-magnitude neoantigen-specific and long-lived polyfunctional CD8 + T cells in pancreatic cancer, resulting in a longer recurrence-free survival [16]. To date, mRNA vaccines have made notable achievements in the field of cancer treatment.

However, mRNA vaccines still face several challenges. The primary concern is their instability and inability to penetrate the physiological barriers in human body, which prevents them from reaching target cells [17]. In the human body, mRNAs are susceptible to degradation by RNases or recognition and phagocytosis by macrophages or dendritic cells (DCs) in the liver [1]. While the naked mRNA can still be taken up by the cell, the process is too inefficient. To increase effectiveness, repeated administrations are required [17]. Nevertheless, excessive amounts of drug can lead to immune-related adverse reactions [18]. In addition, the mRNA vaccine (BNT162b2) administered by intramuscularly injection was mainly distributed in the site of injection and the liver, resulting in reversible liver damage in animals [19]. Therefore, it is challenging to achieve specific organ targeting for mRNA cancer vaccines.

To cope with these challenges, the design and selection of adjuvants is crucial. Adjuvants have the ability to enhance body’s immune response, optimize drug delivery routes, reduce drug toxicity, and enhance drug efficacy by precisely targeting and reducing the total drug volume. However, the definition of adjuvant remains controversial. According to the traditional view, an mRNA vaccine comprises three components: mRNA sequence containing antigen, DDS or vector, and adjuvant. Adjuvant is an immunostimulant that is added in addition to a vector or DDS to non-specifically enhance the body’s specific immune response to the antigen [7]. Conversely, some scholars argue that adjuvants should include DDS in addition to traditional adjuvants [20]. This is because some self-adjuvant delivery materials, such as mesoporous silicon rods, have the ability to enhance the strength, breadth and durability of the immune response itself [21]. In this review, we will discuss immunostimulants or DDS that have an immunopotentiation effect on mRNA vaccines, all of which will be considered as adjuvants. The lack of a systematic overview and summary of the mechanism of action, combined with the complexity of the mechanism and the broad definition of adjuvant, has caused inconvenience and confusion for researchers in designing appropriate vaccine adjuvants [20]. To aid researchers in comprehending adjuvants of mRNA vaccines, this review will describe the design strategies for adjuvants, introduce their mechanisms, and summarize the limitations and side effects of existing adjuvants, and provide prospects for future improvement.

### Design strategies for immunostimulants

**Fig. 2:**
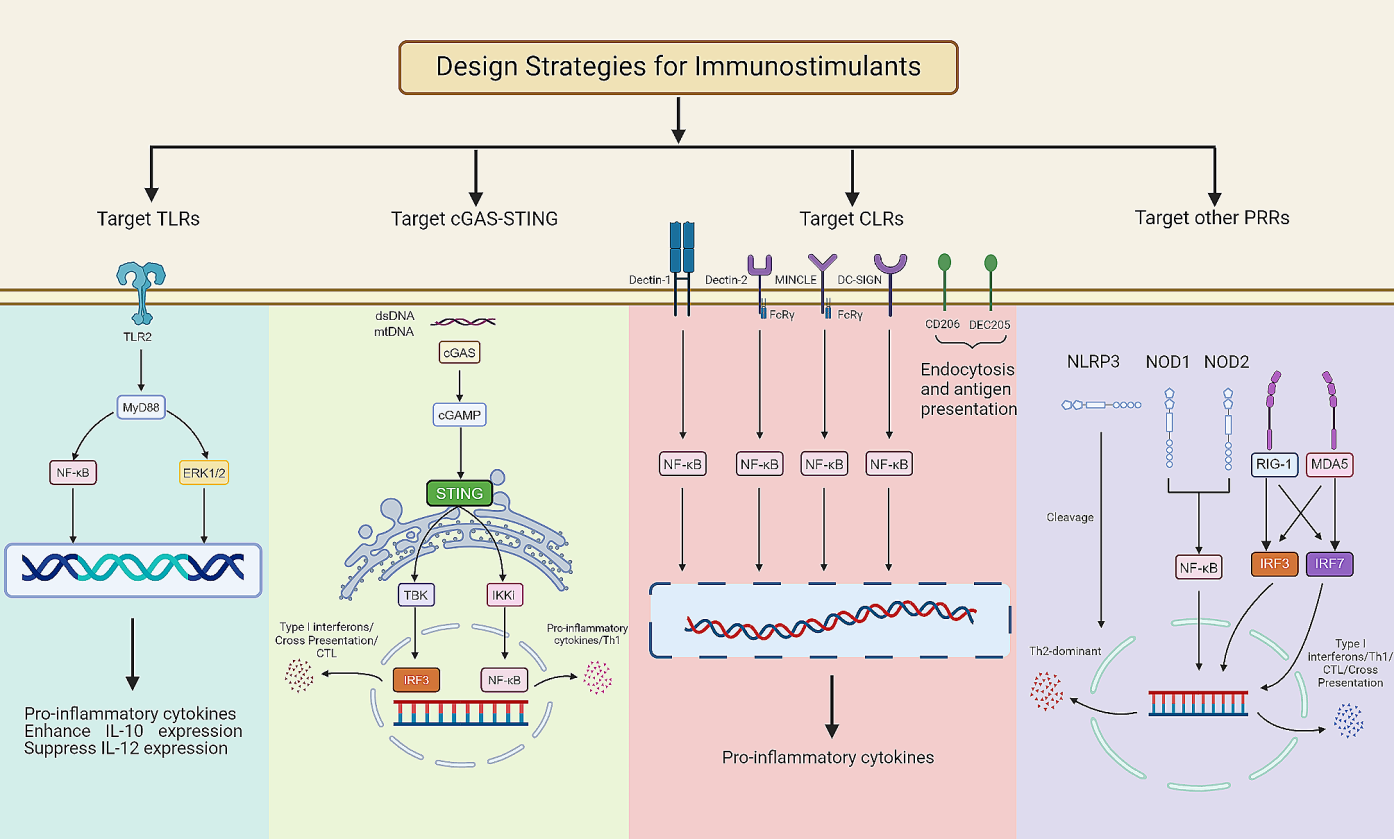
The design strategies for immunostimulants. Various types of immunostimulants activate different PRRs, leading to the secretion of various cytokines and inducing diverse adaptive immune responses. Immunostimulants activate TLRs, cGAS-STING, CLRs, other PRRs, or directly release cytokines to induce and modulate adaptive immune responses. Binding to TLRs heterodimers initiates MyD88 pathway and activated NF-κB and ERK1/2 to enhance pro-inflammatory cytokines. The mtDNA and dsDNA initiates the conversion of cGAS into cGAMP and consequently activates STING to release TBK to activates IRF 3 and IKKi to activates NF-κB. Finally, IRF 3 induces type 1 interferons, cross presentation and CTL and NF-κB induces pro-inflammatory cytokines and activate Th1 cells. Targeting to most CLRs activated NF-κB to enhance pro-inflammatory cytokines. Notably, targeting to CD205 and CD206 can enhance endocytosis and antigen presentation. Common immunostimulants targeting to NLRs (NOD 1 and NOD 2) activated NF-κB ultimately produces a predominantly Th2-type of immune response. Alternatively, immunostimulants targeting to MDA5 and RIG-I activate IRF 3 and IRF 7 respectively. At length, IRF 3 and IRF 7 induce type 1 interferons, cross presentation, CTL and activate Th1 cells

Immunostimulants, often recognized as danger signal molecules, function as pathogen-associated molecular patterns (PAMPs), damage-associated molecular patterns, or their mimics. These substances are pivotal in triggering the innate immune response. They achieve this by targeting pattern recognition receptors (PRRs) on APCs. Upon activation, APCs undergo a maturation process during which their antigen phagocytic activity ceases, and their capability to present antigens is enhanced. Concurrently, these matured APCs express higher levels of co-stimulatory signals and cytokines, which are crucial for initiating and amplifying adaptive immune responses [22]. Currently, the design strategies for immunostimulants are targeting different PRRs to lead different cytokine secretion [23]. Based on the different targeting pathway, there are four dominant design strategies for immunostimulants. Besides, there is a special design strategy for immunostimulants, which is using cytokines as immunostimulants (Fig. 2).

#### Targeting TLRs pathway

Immunostimulants can enhance antigen presentation and upregulate costimulatory signals and cytokine expression by targeting Toll-like receptors (TLRs) on APCs, ultimately enhancing the adaptive immune response [24–28]. One classical mechanism of action for immunostimulants involves their binding to TLRs heterodimers, specifically TLR2/1 or TLR2/6. This interaction initiates signaling through the myeloid differentiation primary response 88 (MyD88) pathway. Subsequent to this signaling event, the transcription factor nuclear factor kappa-light-chain-enhancer of activated B cells (NF-κB) is activated. NF-κB activation leads to the production of pro-inflammatory cytokines, which play a critical role in the differentiation of naive T cells into T helper 1 cells [29, 30]. Simultaneously, the activation of the extracellular signal-regulated kinase 1/2 (ERK1/2) pathway enhances the signaling cascade, leading to increased expression of the c-Fos protein. This increase in c-Fos levels plays a pivotal role in modulating cytokine expression; specifically, it enhances the production of interleukin-10 (IL-10) and suppresses the expression of interleukin-12 (IL-12). This drives the conversion of naive T cells into Th2-type cells [31]. Consequently, immunostimulants targeting TLR2 primarily induce Th2-type adaptive immune response. Here are some common immunostimulants that target TLRs: lipopolysaccharide (LPS), monophosphoryl lipid (MPL), cytosine phospho-guanosine oligonucleotides [CpG (TLR9a)] and R848. LPS, a potent TLR4 agonist, is a natural immune adjuvant from the outer membrane of Gram-negative bacteria [32]. MPL contains the adjuvant active principle of LPS (lipid A) [33]. CpG (TLR9a), an agonist of TLR9, has been widely used as an adjuvant in mRNA cancer vaccine. R848 is recognized by TLR7 and TLR8. The immune cells like monocytes and macrophages are activated by TLR7 and TLR8 and then secrete cytokines to mediate innate and adaptive immune responses [34].

#### Targeting cGAS-STING pathway

The cyclic guanosine monophosphate (GMP)-adenosine monophosphate (AMP) synthase (cGAS) functions as a cytoplasmic DNA sensor that is activated by the presence of double-stranded DNA (dsDNA) and mitochondrial DNA (mtDNA). Upon activation by such DNA, cGAS catalyzes the conversion of cytoplasmic AMP and GMP into cyclic guanosine monophosphate-adenosine monophosphate (cGAMP) [35]. Subsequently, cGAMP aggregates and activates STING through conformational change, which then activates NF-κB and interferon regulatory factor 3 (IRF 3), promoting the production of type I interferons and pro-inflammatory cytokines. Finally, the APCs will have a terrific ability to present or cross-present antigens [36, 37]. Immunostimulants that target the cGAS-STING pathway include nucleotide and non-nucleotide small molecule agonists. The former are mainly natural ligand molecules which based on cyclic dinucleotides. For instance, the cyclic dimeric adenosine monophosphate, cyclic dimeric guanosine monophosphate, 3’,3’-cGAMP, and 2’,3’-cGAMP [38]. Examples of common non-nucleotide small molecule agonists include CF 501 and DMXAA [39, 40].

#### Targeting CLRs pathway

The C-type lectin receptors (CLRs) superfamily comprises various receptors, such as MINCLE, DC-SIGN, Dectin-1, Dectin-2, CD205, CD206, and others. CLRs are primarily located on cell membranes and act as antigen receptors for capturing and presenting antigens [41, 42]. In most instances, immunostimulants that have a carbohydrate structure can activate the CLRs and stimulate the APCs to initiate the internalization, presentation and processing of antigens, thereby enhancing the adaptive immune response [43, 44]. Comparatively, there has been less research on the potential of immunostimulants targeting CLRs pathway. However, it has been found that fungal mannans can act as immune adjuvants, which can elicit a potent antigen-specific neutralizing antibodies to increase the immune response and can be harnessed for vaccine [45]. Immunostimulants that targeted CLRs pathway has a great deal of untapped potential.

#### Targeting other PRRs

In addition to the three major pathways mentioned above, the nucleotide-binding oligomerization domain-like receptors (NLRs) family which includes nucleotide-binding oligomerization domain 1 (NOD1), nucleotide-binding oligomerization structural domain 2 (NOD2), and NOD-like receptor thermal protein domain associated protein 3 (NLRP 3), can also be targeted [46]. Common immunostimulants that target NLRs are muramyl dipeptide and complete Freund’s adjuvant, which ultimately produces a predominantly Th2-type of immune response [28]. Furthermore, retinoic acid-inducible gene I-like receptors family can also be targeted as they primarily recognize RNA. The main members of this family are melanoma differentiation-associated gene 5 (MDA5) and retinoic acid-induced gene I (RIG-I) [47].

#### Cytokine immunostimulant

In addition to the immunostimulants mentioned above, there is a distinct category of immunostimulants called cytokines. Cytokines are small soluble polypeptide proteins secreted by immune and non-immune cells under certain conditions. They play a regulatory role intercellularly and intracellularly. Their effectiveness as adjuvants highly depends on the dose, form, route of administration, and the type of co-administered vaccine [48]. Interleukin-2 (IL-2) and granulocyte-macrophage colony stimulating factor (GM-CSF) are two mature cytokine immunostimulant. GM-CSF promotes the maturation and activation of APCs and IL-2 enhances immune response of T cells [49]. Interferon-alpha (IFN-alpha) and tumor necrosis factor (TNF) are also being investigated for their potential adjuvant effects. They enhance the immunoregulatory function of natural killer cells, promote the differentiation of T lymphocytes and play a broad up-regulatory role in the body’s immune response [50].

Application of immunostimulant in mRNA cancer vaccines.

The current applications of immunostimulant in mRNA cancer vaccines are as follows: (1) Protamine. Arginine-rich protamine peptides have been demonstrated to form a complex with mRNA, subsequently activating TLR7/8 pathways to elicit T-cell and B-cell-dependent immune responses against non-small-cell lung cancer, prostate cancer, and melanoma [51–54]. (2) DP7. The cationic peptide DP7 with cholesterol-modified (VQWRIR-VAVIRK) activates the TLR2-MyD88-IKK-NF-κB pathway and enhance the immune responses stimulated by the mRNA cancer vaccine. Notably, DP7 has been identified as an effective immunostimulant for personalized mRNA cancer vaccines [55]. (3) R848. The TLR7/8 agonist R848 modified with palmitic acid (C16-R848) has been demonstrated to effectively activate the adaptive immune response and to enhance the delivery efficiency of the mRNA cancer vaccine in prostate and lymphoma tumor model mice [56]. (4) α-galactosylceramide (α-GC). The α-GC is a glycolipid antigen that can be presented in the CD1d, the MHC-I-like molecule on APCs, to stimulate invariant natural killer T cells and evoke pluripotent innate and adaptive antitumor immune response in lymphoma animal models [57].

### Design strategies for self-adjuvant drug delivery systems for mRNA vaccines

**Fig. 3:**
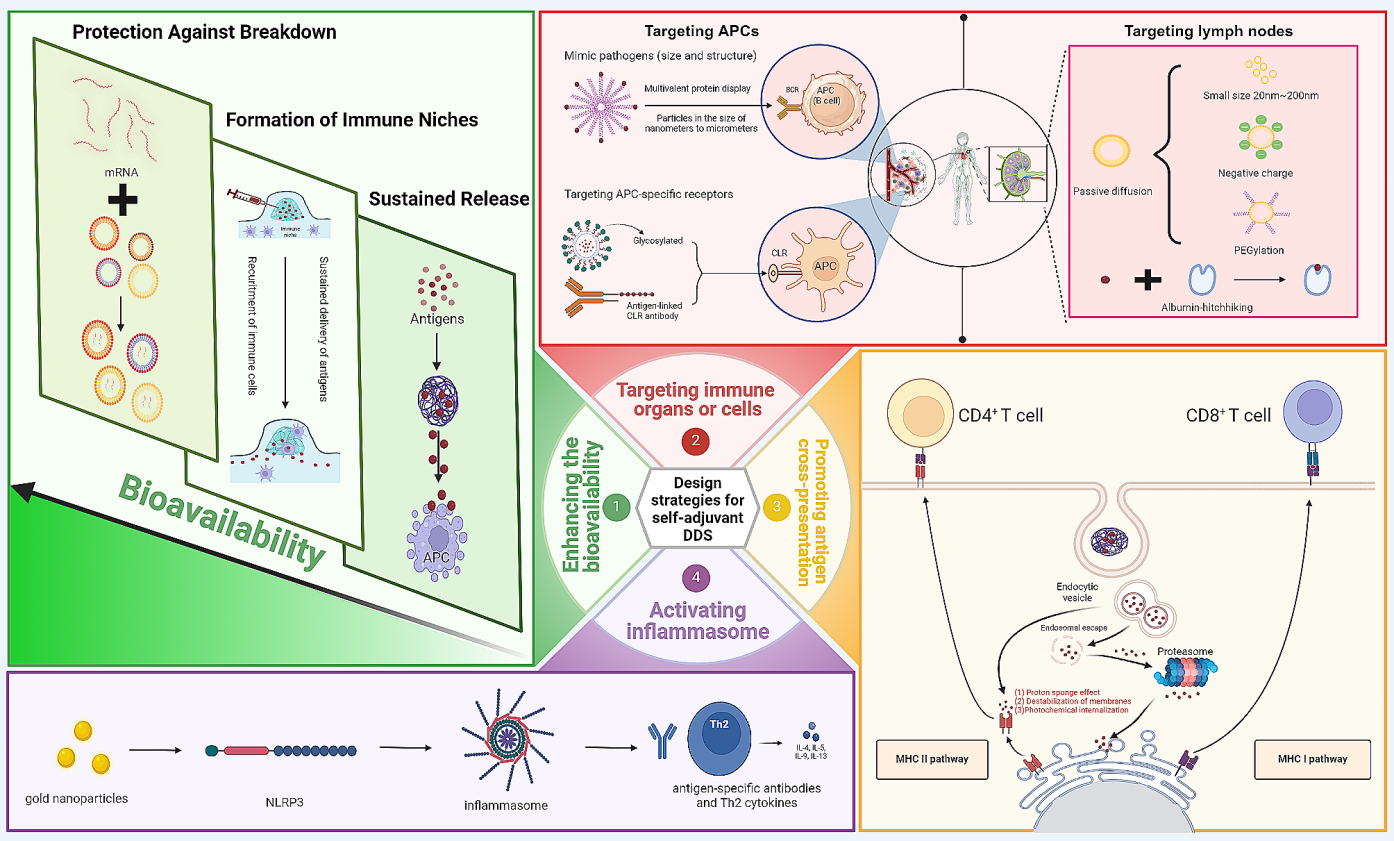
The design strategies for self-adjuvant drug delivery systems. Self-adjuvant delivery systems increased antigen presentation to enhance adaptive immune responses by enhancing the bioavailability of antigens; targeting immune organs or cells; promoting antigen cross-presentation; and activating inflammasome. Enhancing the bioavailability of antigens can be achieved by sustained releasing antigens, formatting immune niches, and protecting antigens from breakdown. Targeting APCs can be achieved by using nanoscale materials, constructing highly ordered and repetitive spatial structures to mimic pathogens and targeting specific receptors on APCs. Targeting lymph node can achieved through the design of suitable dimensions (20 to 200 nm), surface properties (negative charge and hydrophobicity) and albumin-hitchhiking. Promoting antigen cross-presentation can be enabled in three main ways, proton sponge effect, destabilization of membranes and photochemical internalization

The definition of DDS is carrier materials that load antigen and increase the ability of APCs to uptake and present antigen [20]. The main function of DDS for mRNA vaccines is to aid in antigen presentation by assisting mRNAs in crossing the three barriers of the body: the extracellular barrier, lysosomal escape, and intracellular immunity. This results in an increase in the antigenic signals on the surface of APCs [1]. Notably, the precise DDS for mRNA vaccines can reduce the toxicity of the vaccine, reduce the total amount of drug, and increase the efficacy of the vaccine. Currently, there are two main types of delivery vectors: viral vectors and non-viral vectors. Although viral vectors have the advantage of high transfection efficiency, the safety concerns remain a significant issue. The enthusiasm for viral vector research has largely waned after two clinical trials in which the use of viral vectors resulted in the deaths of volunteers [58, 59]. Attention has shifted to non-viral vectors due to the stagnation of viral vector research. Non-viral vectors, with their low toxicity and low immunogenicity, have become one of the hottest research directions at present. Nevertheless, drugs delivered by non-viral vectors also have the disadvantage of low escape efficiency in nuclear endosomes or lysosomes and weak ability to target to cells, tissues, and organs [60, 61]. There is an urgent need for novel non-viral DDS for mRNA vaccines to overcome these difficulties. There are four common strategies (Fig. 3):

#### Enhancing the bioavailability of antigens

(1) **Sustained Release**: By prolonging the presence of antigens within the immune system through their sustained release, there is a prolonged opportunity for immune system interaction. This method ensures that antigens are continuously available to stimulate an immune response [62]. (2) **Formation of Immune Niches**: Creating immune niches at the site of injection can recruit additional immune cells. This influx of immune cells enhances antigen uptake and activates the adaptive immune response, thereby stimulating the release of cytokines and chemokines. This process intensifies the immune system’s engagement with the antigen [63]. (3) **Protection Against Breakdown**: Protecting antigen from breakdown by mRNA enzymes and slowing down the antigen digestion process are crucial, which is one of the benefits of lipid nano-particle (LNP) [4, 64, 65].

#### Targeting immune organs or cells

Targeting APCs in the immune microenvironment can enhance immune presentation and phagocytosis. Several methods can be employed to achieve this: (1) Using microscale or nanoscale materials to adjust the dimension of antigen to mimic pathogens [66]. (2) Constructing highly ordered and repetitive spatial structures similar to those inherent in pathogens allows the immune system to recognize and respond to these structural features with greater sensitivity [67]. Furthermore, these structures can facilitate the co-aggregation of B cell receptor (BCR) and the eventually produce high-affinity antibodies and memory B cells [68, 69]. (3) Targeting the specific receptors on APCs, such as Fc receptors [70–73].

Lymph node metastasis is a significant prognostic factor that signals a worse prognosis and reflects the necessity of systemic therapy in the majority of cancer patients [74]. For patients with oral squamous cell carcinomas, the five-year survival rate can decline to below 20% when lymph node metastasis occur [75]. Consequently, targeting the mRNA vaccines to lymph nodes is an ideal design strategy for DDS. The precise delivery of mRNA vaccine to lymph nodes can change the pharmacokinetics, activate a long-lasting and potent immune response, and reduce undesired systemic toxicity and side effects [76, 77]. In addition, targeting lymph nodes can significantly augment the innate immune response, particularly by activating macrophages within the lymph nodes, which in turn enhances the anti-tumor efficacy of mRNA vaccines [78]. The high anti-tumor efficacy of the lymph node-targeting DDS demonstrates considerable potential as a design strategy for mRNA vaccines [77].

Effective strategies to achieve this include: (1) Designing a DDS for mRNA vaccines of suitable dimensions (20 to 200 nm) and surface properties (net negative charge and hydrophobicity) that relies on passive diffusion to enter the afferent lymphatics and subsequently enter lymph nodes [79–81]. (2) Albumin-hitchhiking, which exploits the ability of endogenous albumin to circulate in the lymphatic system. Binding the antigen to endogenous albumin, thereby antigen is transported to the lymph nodes via the albumin train [82, 83]. Notably, this method is found to be highly effective in inhibiting the growth of primary or metastatic tumors in mice [84].

Alternatively, in LNP, there is a special target needs to be considered cautiously, the non-liver tissues target [85]. LNP is a mature technique for the delivery of genetic medicines. However, its therapeutic application is limited due to the liver accumulation. The apolipoprotein E in serum binds to LNP and causes mRNA to preferentially enter the liver, which produces enzymes that interfere with the effectiveness of the mRNA vaccine, preventing the LNP@mRNA from achieving its full potential [86]. In order to address liver accumulation, the addition of the selective organ targeting (SORT) lipids can achieve specific targeting of organs such as the liver, lungs, spleen, etc., thus enabling non-liver tissues target [85].

#### Promoting antigen cross-presentation

In most cases, exogenous antigens are just internalized by APCs and only presented to CD4^+^ T cells by major histocompatibility complex II (MHC II) molecules, without any cross-presentation which is the process by which exogenous antigens are presented to CD8 + T cells by major histocompatibility complex I (MHC I) molecules [87, 88]. This type of presentation elicits a weak immune response. To increase the strength of the immune response, some DDS for mRNA vaccines have enabled antigen cross-presentation by facilitating the escape of antigens from lysosomes or endosomes [20]. Cross-presentation can be enabled in three main ways: (1) Proton sponge effect. When some DDS for mRNA vaccines containing protonable amine groups are internalized by the APCs, substantial protons are absorbed by the APCs. To neutralize the acidic environment of the lysosome or endosomal of APCs, chloride ions and water will flow from the cytoplasm into endosomes or lysosomes in large amounts, which causes swelling and rupture of the endosomes. Subsequently, the antigens are released into the cytoplasm, which facilitates the cross-presentation of the antigen by MHC I molecules [89]. (2) Binding or fusing to the membranes of endosomal or lysosomal. This process destabilizes the endosomal/lysosomal membrane, releasing the antigen into the cytoplasm, which facilitates the cross-presentation of the antigen by MHC I molecules [90]. (3) Photochemical internalization release technology. This is an emerging technology that uses photosensitizers to release antigens into the cytoplasm through light-induced disruption of endosomal membranes [91–93].

#### Activating inflammasome

Inflammasome, multi-protein complexes assembled with the participation of PRRs, is an important component of the innate immune system [94]. Gold nanoparticles, one of the most mature inorganic nano-drug delivery systems, can promote the production of antigen-specific antibodies and Th2 cytokines through the activation of NLRP3 inflammasome, in addition to the protection of antigens from hydrolysis by mRNA enzymes and targeting to the lymph nodes mentioned above [95].

#### Application of self-adjuvant DDS in mRNA cancer vaccines

Currently, the applications of self-adjuvant DDS in mRNA cancer vaccines are listed as follows: (1) LNP. The use of endogenously LN-targeting LNP can improve the effectiveness of mRNA vaccine by stimulating robust humoral responses and T follicular helper cell [96]. The 113-O12B is an effective LN-targeting DDS for mRNA cancer vaccines and can improve the effectiveness of anti-tumour treatment [77]. The BNT-113, an mRNA vaccine encapsulated within LNP, has demonstrated encouraging efficacy against head and neck cancer. It is currently undergoing phase II clinical trials (NCT04534205) [97]. In addition, the mRNA-4157 is a personalized mRNA vaccine encapsulated in LNP too. It can encode multiple neoantigens, thereby inducing neoantigen-specific T cells and eliciting anti-tumor immune responses in patients with head and neck cancer [98]. Furthermore, loading comb-structured mRNA, which consists of antigen-producing single-stranded mRNA, and adjuvant short double-stranded RNA, onto LNP enables immunostimulation in different formulations of mRNA cancer vaccines [99]. The mRNA vaccine combining all-trans-retinoic acid with LNP has shown significant tumor inhibition effects in animal model for the treatment of orthotopic colorectal tumors [100]. (2) Polyguanidine (PolyGu). Branched PolyGu nanovaccines are used to integrate immunostimulant functions into the DDS, resulting in self-adjuvating PolyGu nanovaccines. It can effectively stimulate and promoted the maturation of DCs through TLR4 and NLRP3 pathways, and exhibited strong immune activity in vivo. In addition, PolyGu can improve the delivery efficiency of mRNA as a DDS and effectively suppress tumour growth, thereby prolonging the survival of mice [101]. . (3) Self-assembled RNA origami (RNA-OG). The RNA-OG nanostructure functions as a TLR 3 agonist and is a suitable DDS for mRNA cancer vaccines due to its versatility in modification and robust synthesis. Studies have shown that the assembled RNA-OG-peptide nanovaccines induce DCs maturation, mobilize tumor-specific CD8 + T cell responses, and reduce tumor-mediated immunosuppression [102]. In the field of colorectal cancer, the lantern-shaped flexible origami can compress mRNA to nanoscale, thereby promoting its endocytosis by cells and improving translation efficiency. The mRNA nano-lantern facilitates the overexpression of Smad4, a tumor suppressor gene, in orthotopic colorectal tumor models, effectively inhibiting their growth [103]. This origami strategy offers a competitive DDS for mRNA-based therapies in the treatment of colorectal cancer. (4) Outer membrane vesicles (OMVs). OMVs contain numerous PAMPs that can effectively stimulate the innate immune system, facilitating T cell activation and antigen presentation. OMVs with surface decoration of lysosomal escape protein listeriolysin O and RNA binding protein, L7Ae, (OMV-LL) can be cross-presentation and OMV-LL mRNA significantly inhibits the progression of melanoma [104]. (5) Porous silica nanoparticles. This mRNA DDS is based on polyethylenimine-modified porous silica nanoparticles. It promotes effective antitumor immunity without evidence of systemic toxicity and off-target translation of mRNA [105]. It also inhibited distant metastatic tumors and improved anti-tumor responses in murine cancer models. (6) Iron oxide. This is a magnetically multi-functional RNA-loaded liposome that is capable of generating a robust anti-cancer immune response. Comparing to electroporation, this mRNA DDS activates DCs more effectively, resulting in superior tumor growth inhibition in animal models [106].

### Deficiencies of the adjuvants of mRNA cancer vaccines

Current clinical trials of mRNA vaccines have shown that adverse reactions such as fatigue, pain at the injection site, myalgia, narcolepsy and neurological side effects can be triggered by the mRNA vaccines [107–109]. Notably, there are also a number of adverse reactions and limitations associated with adjuvants that need to be considered. Immune-related adverse event is one of the most notable side effects. Reports of anaphylactic reactions induced by mRNA vaccines are still being received [110]. At the injection site, adjuvanted vaccines are more reactogenic than non-adjuvanted vaccines, which can cause immune-related adverse events such as anaphylaxis. However, the symptoms are typically mild to moderate and of short duration [18]. Notably, immune activation and cytotoxicity may be triggered when injected above a certain dose of mRNA or ionizable lipids. Ultimately, allergic reactions and even cytokine storms may be triggered [111]. In preclinical mRNA vaccine studies, LNP were found to be highly inflammatory in mice, triggering a severe inflammatory response [112]. The ionizes lipids SM-102, which is ionizable and used in vaccines, may cause vaccinator to experience adverse effects such as nausea [113]. Besides, in PEGylated lipids, PEG may cause allergic reactions [114].

**Fig. 4:**
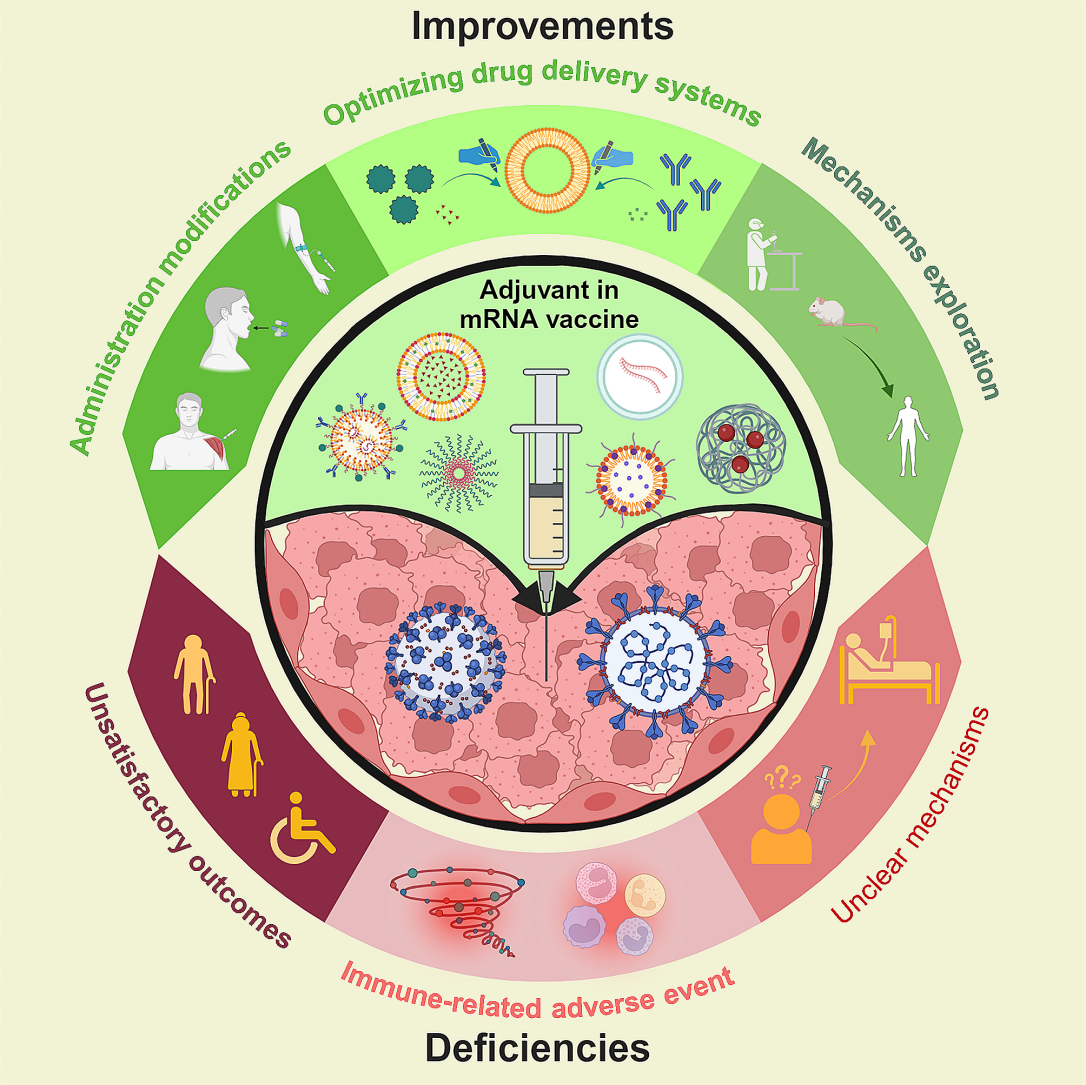
The deficiencies of mRNA adjuvants and the prospects for improvement. The immune-related adverse event, unsatisfactory outcomes in old age group and unclear mechanism of adjuvants are three main deficiencies of the mRNA adjuvants. Alternatively, optimizing drug delivery systems, improving route of administration and further explored the mechanisms of adjuvant are three main prospects for the improvement of mRNA adjuvants

In addition, the unsatisfactory outcomes and unclear mechanisms of most approved adjuvants cannot be ignored. Comparatively, the mRNA vaccines have low immunogenicity and produce weak and short-lived immunity in the body [115]. The vaccine’s protective effect is low until two vaccinations were completed. Additionally, the effectiveness of vaccine declined with age, and is ineffective in the old age group [116]. Alternatively, the development of mRNA adjuvants is still in its early stages, and the construction of DDS for mRNA vaccines is immature. The mechanisms of many adjuvants remain unclear. Some basic problem such as the endosomal escape process remain unclear too [117]. Unclear mechanisms of adjuvants can result in improper use of adjuvants and the emergence of side effects. The deficiencies of the adjuvants of mRNA vaccines are summarized in Fig. 4.

### Prospects for improvement

#### Further explored the mechanisms of adjuvant

The formation of an immune niche (antigen depot), as mentioned above, is traditionally thought to be one of the important mechanisms of adjuvants. Nevertheless, with the deepening of researches, some researchers found that the removal of the “immune niche” after aluminum adjuvant administration didn’t substantial reduce the generation of B cell responses and antigen-specific T [118]. This suggests that the formation of an immune niche may not be the key mechanism of action of aluminum adjuvants. From this, we can further hypothesize whether other adjuvant systems are also like the aluminum adjuvant system. For more rational use of adjuvants and the development of new adjuvants, the mechanism of adjuvants should be better explored. Alternatively, the source of the adjuvant effect requires further researched. Immunostimulants and self-adjuvanted DDS for mRNA vaccines are not the only methods for achieving adjuvant effects. Editing the RNA itself can also produce adjuvant effect. However, the mechanism behind it requires further investigation [99].

#### Optimizing drug delivery systems

Using LNP as an example, DDS optimization can be achieved by optimizing lipid structure and targeted molecular. It has been shown that by modifying the lipid structure of lipid nanoparticles, including tail length, linkages and amine heads, and by optimizing the proportion of different lipid components of lipid nanoparticle formulations, lymph node-targeted delivery can be achieved to enhance vaccine immunity effects [77, 119]. Notably, the modification of targeting molecules on the surface of LNP or alteration of the properties of the LNP can enhance the efficacy of vaccine by targeting LNP delivery to specific cells or organs. For example, mannosylation of lipid nanoparticles can enhance the uptake of APCs [120]. Modulation of the surface charge of RNA-lipid complexes enables precise and efficient targeting of DCs [121]. In addition, for non-liver tissues target, permanent cationic SORT lipids (EPC, DDAB, and DOTAP) can be applied to shift tissue tropism from the liver to the lung [13]. Alternating the alkyl length of a lipid or changing the intermediate connecting group of LNP from an ester bond to an amide bond in the tail can also change the organ targeting of LNP to liver or lung [122, 123].

In addition to the existing DDS for mRNA vaccines, there are many novel DDS besides liposomes waiting to be discovered. For instance, engineered extracellular vesicles (EVs) with pathogen proteins is a promising alternative to LNP-mRNA vaccines. With their ability to naturally target and transport bioactive molecules, these engineered EVs are expected to overcome the problems of complex and non-continuous manufacturing processes and expensive materials involved in mRNA vaccine production [124].

#### Improving route of administration

Route of administration can greatly influence expression, kinetics organ distribution, and therapeutic outcome of LNP-mRNA vaccine [125, 126]. Intravenous administration, as mentioned above, has the potential to enhance the immune response to mRNA vaccines, although issues of targeting non-liver tissues remain to be addressed [121]. Nevertheless, topical administration also has its own unique local therapeutic effect, allowing for the supplementation of therapeutic proteins in specific tissues such as brain, heart, eyes [127–129]. In addition, there are other routes of administration such as the intranasal route [130]. In order to better exploit the advantages of different routes of administration, a comprehensive decision on which route to use needs to be made after careful consideration of factors such as the nature of the nanoparticles and the therapeutic indications. The prospects for improving of mRNA vaccines are summarized in Fig. 4.

## Conclusion

Although there is still a long way to go in optimizing mRNA vaccines, their excellent biocompatibility, high tissue penetration, high nucleic acid encapsulation efficiency, low occurrence of off-target effects, cytotoxicity, and immunogenicity have made mRNA cancer vaccine one of the hottest research areas in vaccines today. Further researches are required to elucidate the mechanism of adjuvants and to optimize the design strategy and DDS for mRNA vaccines. Additionally, the development of new DDS for mRNA cancer vaccines and personalized mRNA cancer vaccines are promising avenues for future investigations. With the continued refinement of next-generation adjuvants, this new technology will help solve problems that traditional small-molecule and antibody therapies cannot, providing more effective and longer-lasting therapeutic outcome in the treatment of a wide range of diseases, including tumors, and improving healthcare in the near future.

## Data Availability

No datasets were generated or analysed during the current study.

## Abbreviations

DDS: drug delivery systems
COVID-19: coronavirus disease 2019
APCs: antigen-presenting cells
DCs: dendritic cells
PAMPs: pathogen-associated molecular patterns
PRRs: pattern recognition receptors
TLRs: Toll-like receptors
MyD88: myeloid differentiation primary response 88
NF-κB: nuclear factor kappa-light-chain-enhancer of activated B cells
ERK1/2: extracellular signal-regulated kinase 1/2
IL-10: interleukin-10
IL-12: interleukin-12
LPS: lipopolysaccharide
MPL: monophosphoryl lipid
CpG (TLR9a): cytosine phospho-guanosine oligonucleotides
dsDNA: double-stranded DNA
mtDNA: mitochondrial DNA
GMP: guanosine monophosphate
AMP: adenosine monophosphate
cGAS: cyclic GMP-AMP synthase
cGAMP: cyclic guanosine monophosphate-adenosine monophosphate
IRF 3: interferon regulatory factor 3
TBK: TANK-binding kinase
IKKi: inhibitor of NF-kappaB kinase
CTL: cytotoxic T lymphocyte
CLRs: C-type lectin receptors
NLRs: nucleotide-binding oligomerization domain-like receptors
NOD1: nucleotide-binding oligomerization domain 1
NOD2: nucleotide-binding oligomerization structural domain 2
NLRP 3: NOD-like receptor thermal protein domain associated protein 3
MDA5: melanoma differentiation-associated gene 5
RIG-I: retinoic acid-induced gene I
IRF 7: interferon regulatory factor 7
IL-2: interleukin-2
GM-CSF: granulocyte-macrophage colony stimulating factor
IFN-alpha: interferon-alpha
TNF: tumor necrosis factor
α-GC: α-galactosylceramide
PLGA: poly(lactic-co-glycolic acid)
LNP: lipid nano-particle
BCR: B cell receptor
SORT: selective organ targeting
MHC II: major histocompatibility complex II
MHC I: major histocompatibility complex I
PolyGu: polyguanidine
RNA-OG: RNA origami
OMVs: outer membrane vesicles
OMV-LL: OMVs with surface decoration of listeriolysin O and L7Ae
EVs: extracellular vesicles
